# Delirium is significantly associated with hospital frailty risk score derived from administrative data

**DOI:** 10.1002/gps.5872

**Published:** 2023-01-22

**Authors:** Zhiying Lim, Natalie Ling, Vanda Wen Teng Ho, Nachammai Vidhya, Matthew Zhixuan Chen, Beatrix Ling Ling Wong, Shu Ee Ng, Diarmuid Murphy, Reshma Aziz Merchant

**Affiliations:** ^1^ Division of Geriatric Medicine Department of Medicine National University Hospital Singapore Singapore; ^2^ Department of Orthopaedic Surgery National University Hospital Singapore Singapore; ^3^ Department of Medicine Yong Loo Lin School of Medicine National University of Singapore Singapore Singapore

**Keywords:** cost, delirium, frailty, outcomes

## Abstract

**Objectives:**

Delirium is highly prevalent in hospitalised older adults, under‐diagnosed and associated with poor outcomes. We aim to determine (i) association of frailty measured using Hospital Frailty Risk Score (HFRS) with delirium, (ii) impact of delirium on mortality, 30‐days readmission, extended length of stay (eLOS) and cost (eCOST).

**Methods:**

Retrospective cohort study was conducted on 902 older adults ≥75 years discharged from an academic tertiary hospital between March and September 2021. Data was obtained from hospital administrative database.

**Results:**

Delirium was prevalent in 39.1%, 58.1% were female with mean age 85.3 ± 6.2 years. Patients with delirium were significantly older, had higher HFRS, pneumonia, urinary tract infection (UTI), E.coli and *Klebsiella* infection, constipation, dehydration, stroke and intracranial bleed, with comorbidities including dementia, diabetes, hypertension, hyperlipidaemia and chronic kidney disease. In‐hospital mortality, 30‐days mortality, 30‐days readmission, median LOS and cost was significantly higher. Delirium was significantly associated with at least intermediate frailty (OR = 3.52; CI = 2.48–4.98), dementia (OR = 2.39; CI = 1.61–3.54), UTI (OR = 1.95; CI = 1.29–2.95), constipation (OR = 2.49; CI = 1.43–4.33), *Klebsiella* infection (OR = 3.06; CI = 1.28–7.30), dehydration (OR = 2.01; CI = 1.40 ‐ 2.88), 30‐day mortality (OR = 2.52; CI = 1.42–4.47), 30‐day readmission (OR = 2.18; CI = 1.36–3.48), eLOS (OR = 1.80; CI = 1.30–2.49) and eCOST (OR = 1.67; CI = 1.20–2.35).

**Conclusions:**

Delirium was highly prevalent in older inpatients, and associated with dementia, frailty, increased cost, LOS, 30‐day readmissions and mortality. Hospital Frailty Risk Score had robust association with delirium and can be auto‐populated from electronic medical records. Prospective studies are needed on multicomponent delirium preventive measures in high‐risk groups identified by HFRS in acute care settings.

## Key points

- Delirium is highly prevalent in older inpatients, and associated with at least intermediate frailty on the Hospital Frailty Risk Score (HFRS), dementia, increased cost, length of stay (LOS), 30‐day readmissions and mortality.
- Delirium is strongly associated with poor outcomes and robustly associated with HFRS, which can be auto‐populated from electronic medical records for widespread implementation in healthcare systems to identify vulnerable older adults at risk of delirium.

## 1 INTRODUCTION

The word ‘delirium’ was first coined by Aulus Cornelius Celsus (c. 25 BC–c. 50 AD) in the first century AD to describe mental disorders during fever or trauma, and later added non‐febrile causes.^1^ The definition of delirium has evolved over the years as a complex neuropsychiatric syndrome characterised by a disturbance in attention, awareness and cognitive function, with an acute onset and fluctuating course.^2^ The prevalence of delirium amongst hospitalised older adults varies between 20% and 70% depending on underlying clinical condition and illness severity.^3^, ^4^ Delirium has been shown to be strongly and independently associated with poor patient outcomes, including higher in‐hospital and 6‐month mortality, greater nursing time per patient, prolonged LOS, higher hospital costs, higher institutionalisation rates with poor functional and cognitive recovery.^3^, ^5^, ^6^ Despite years of research, delirium remains challenging to define, its pathophysiology poorly understood, and a gold standard diagnostic tool is still yet to exist. Given the heavy financial and healthcare burden associated with delirium, early detection and prevention remains critical in any institutional care setting.^7^

Age, dementia, severe illness, poor vision, urinary catheters, polypharmacy, and low albumin are known risk factors for delirium and in recent years, frailty is increasingly being recognised as a risk factor.^8^, ^9^, ^10^ Frailty is characterised by a state of vulnerability and poor homeostatic capacity to respond to stressors as a result of cumulative physiological decline, resulting in poorer health outcomes.^11^ Various frailty assessment tools have been used in the acute setting including the Frailty phenotype,^12^ deficit‐accumulation frailty index,^13^ Clinical Frailty Scale, and Edmonton Frail scale.^14^ They require direct physical and cognitive assessments which may be resource intensive, which limits the generalizability of use in the acute care setting. Measuring frailty in the acute care setting has been an ongoing challenge. More recent trends have been frailty scores captured from electronic medical records such as the electronic frailty index and the HFRS.^14^, ^15^, ^16^ While HFRS has been associated with mortality, readmission, cost and LOS, to date there are no studies on association of delirium with HFRS. We aim to determine (i) association of frailty measured using HFRS with delirium, and (ii) impact of delirium on in‐hospital mortality, 30‐days mortality, 30‐days readmission, extended length of stay (eLOS) and extended cost (eCOST).

## 2 MATERIALS AND METHODS

### 2.1 Database and study population

A retrospective cohort study was conducted on 902 older adults ≥75 years old who were discharged from Division of Geriatric Medicine between March and September 2021. Existing, de‐identified data was extracted from the hospital's administrative database. Delirium diagnosis was based on either a primary or secondary diagnosis coded under the following International Classification of Disease, 10th revision (ICD‐10) codes: “F051 Delirium superimposed on dementia”, “F058: Other delirium” or “F059: Delirium, unspecified”.

### 2.2 Demographics data

Data was obtained on age, gender, ethnicity, primary and secondary diagnoses, comorbidities, age adjusted Charlson Comorbidity Index, HFRS and serum albumin. The prevalence of 12 common either primary or secondary diagnoses (pneumonia, fragility fractures, acute retention of urine, urinary tract infection (UTI), constipation, hyponatremia, dehydration, stroke, intracranial bleed, acute myocardial infarction, *E. coli* infection and *Klebsiella* infection) in the study group or known to cause delirium in older patients were analysed.

Hospital Frailty Risk Score was initially described by Gilbert et al where a frailty risk score is generated based on ICD‐10 codes.^16^ Frailty is subcategorised based on HFRS scores into low (<5), intermediate (5–15) and high (>15). The role of HFRS in predicting LOS, mortality, adverse events and cost has been validated in multiple patient populations.^14^, ^17^ Age‐adjusted Charlson comorbidity index initially validated to predict mortality is a weighted index of age, number and seriousness of comorbid disease.^18^

### 2.3 Outcome data

Data was collected on LOS, in‐hospital mortality, 30‐days mortality and 30‐days readmission. Cost data obtained included total cost and breakdown by physiotherapy, occupational therapy, dietitian consultation, medication, laboratory and radiology cost. Extended LOS (eLOS) and extended cost (eCOST) were defined as those with above 75th percentile for LOS and cost respectively.

### 2.4 Statistical analysis

IBM SPSS Version 28.0 was used for analysis with statistical significance set at 2 sided 5%. Descriptive analyses were presented as frequencies with percentages for categorical variables and mean with standard deviation or median and interquartile range for continuous variables. Significance by Pearson *χ*2 test for categorical variables, and by Mann–Whitney U test for continuous variables was conducted. Multivariate logistic regression model for clinical binary outcomes were adjusted for age, frailty, dementia, and Charlson comorbidity index. Odds ratios with 95% confidence intervals are presented.

### 2.5 Ethics

The need for informed consent was waived as no direct contact with patients occurred and anonymized data was collected. The study was reviewed and approved by the National Healthcare Group Domain Specific Review Board (Reference: 2021/01116).

## 3 RESULTS

Amongst 902 patients discharged, the prevalence of delirium was 39.1%, with 58.1% females and a mean age of 85.3 ± 6.2 years (Table 1). Patients with delirium were significantly older (86.1 ± 6.3 years vs. 84.1 ± 6.0 years) and had higher prevalence of infections such as pneumonia (37.7% vs. 25.3%), UTI (32.6% vs. 14.0%), *E. coli* infection (17.6% vs. 10.0%) and *Klebsiella* infection (6.2% vs. 1.8%). Conditions such as constipation (13.3% vs. 5.8%), dehydration (39.1% vs. 18.6%), stroke (3.7% vs. 0.5%) and intracranial bleed were also more prevalent in older patients with delirium. Underlying co‐morbidities such as diabetes, hypertension, hyperlipidaemia, and chronic kidney disease were significantly more prevalent in patients with delirium. Dementia was almost 3 times more prevalent in patients with delirium compared to those without (33.4% vs. 12.6%).

**TABLE 1:** Demographics and outcome of delirium as primary or secondary diagnosis

	All	Delirium	No delirium	*p*‐value
	902	353 (39.1%)	549 (60.9%)	
Demographics				
Gender				0.783
Male	378 (41.9)	150 (42.5)	228 (41.5)	
Female	524 (58.1)	203 (57.5)	228 (58.5)	
Age (years)	85.3 ± 6.2	84.1 ± 6.0	86.1 ± 6.3	**0.002**
Ethnicity				0.255
Chinese	743 (82.4)	282 (79.9)	461 (84.0)	
Malay	61 (6.8)	27 (7.6)	34 (6.2)	
Indian	65 (7.2)	33 (9.3)	32 (5.8)	
Others	33 (3.6)	11 (3.1)	22 (4.0)	
Diagnosis				
Pneumonia	272 (30.2)	133 (37.7)	139 (25.3)	**<0.001**
Fragility fracture	44 (4.9)	15 (4.2)	29 (5.3)	0.530
Acute retention of urine	98 (10.9)	47 (13.3)	51 (9.3)	0.063
Urinary tract infection	192 (21.3)	115 (32.6)	77 (14.0)	**<0.001**
Constipation	79 (8.8)	47 (13.3)	32 (5.8)	**<0.001**
Hyponatremia	133 (14.7)	59 (16.7)	74 (13.5)	0.211
Dehydration	240 (26.6)	138 (39.1)	102 (18.6)	**<0.001**
Stroke	16 (1.8)	13 (3.7)	3 (0.5)	**<0.001**
Intracranial bleed	21 (2.3)	12 (3.4)	9 (1.6)	**0.112**
malnutrition	171 (19.0)	86 (24.4)	85 (15.5)	**0.001**
Acute myocardial infarction	78 (8.6)	41 (11.6)	37 (6.7)	**0.015**
*E. Coli* infection	117 (13.0)	62 (17.6)	55 (10.0)	**0.001**
*Klebsiella* infection	32 (3.5)	22 (6.2)	10 (1.8)	**<0.001**
Dysphagia	194 (21.5)	110 (31.2)	84 (15.3)	**<0.001**
Diabetes	364 (40.4)	158 (44.8)	206 (37.5)	**0.031**
Hypertension	490 (54.3)	207 (58.6)	283 (51.5)	**0.040**
Hyperlipidaemia	310 (34.4)	138 (39.1)	172 (31.3)	**0.018**
Dementia	187 (20.7)	118 (33.4)	69 (12.6)	**<0.001**
Chronic kidney disease	336 (37.3)	157 (44.5)	179 (32.6)	**<0.001**
Serum albumin		32.9 ± 5.5	34.6 ± 4.9	**<0.001**
Hospital frailty risk score (median (IQR))	6.1 (10.6)	10.7 (10.5)	3.9 (7.9)	**<0.001**
Low	390 (43.2)	73 (20.7)	317 (57.7)	**<0.001**
Intermediate	366 (40.6)	172 (48.7))	194 (35.3)	
High	146 (16.2)	108 (30.6)	38 (6.9)	
Age adjusted Charlson's comorbidity index (median (IQR))	6.0 (3.0)	6.0 (3.0)	6.0 (3.0)	**<0.001**
Outcomes				
In‐hospital mortality	63 (7.0)	27 (7.6)	36 (6.6)	**0.009**
30‐day mortality	71 (7.9)	47 (13.3)	24 (4.4)	**<0.001**
30 days readmission	98 (10.9)	54 (15.3)	44 (8.0)	**<0.001**
Length of stay				
Mean	6.7 ± 6.3	8.7 ± 7.8	5.4 ± 4.6	**<0.001**
Median (IQR)	5.0 (5.0)	7.0 (7.0)	4.0 (5)	**<0.001**
Cost (total)				
Mean	$5617 ± 4313	$6918 ± $5010	$4780 ± $3560	**<0.001**
Median (IQR)	$4429 ($4116)	$5289 ($4921)	$3816 ($3855)	**<0.001**
Physiotherapy cost				
Mean	$162 ± $188	$203 ± $240	$135 ± $138	**<0.001**
Median (IQR)	$112 ($168)	$112 ($224)	$112 ($140)	**<0.001**
Occupational therapy cost				
Mean	$138 ± $143	$173 ± $172	$115 ± $116	**<0.001**
Median (IQR)	$112 ($112)	$112 ($168)	$84 (112)	**<0.001**
Speech therapist*				
Mean	$49 ± $79	$74 ± $92	$32 ± $64	**<0.001**
Dietitian cost				
Mean	$47 ± $60	$63 ± $65	$37 ± $53	**<0.001**
Median	0 ($100)	$60 ($100)	$0 ($60)	**<0.001**
Laboratory cost				
Mean	$883 ± 600	$1018 ± $654	$797 ± $547	**<0.001**
Median	$730 ($662)	$841 ($719)	$649 ($605)	**<0.001**
Radiology cost				
Mean	$522 ± $723	$559 ± $668	$498 ± $756	0.203
Median	$250 ($653)	$450 ($648)	$147 ($$638)	**<0.001**
Medication cost				
Mean	$375 ± $601	$559 ± $668	$498 ± $756	**0.02**
Median	$260 ($312)	$450 ($648)	$147 ($638)	**<0.001**

*Note*: Bold values are statistically significant.

Serum albumin which is often used as a surrogate for malnutrition and/or illness severity was significantly lower in patients with delirium (32.9 ± 5.5 g/dl vs. 34.6 ± 4.9 g/dl). The HFRS median score was significantly higher in patients with delirium (10.7 IQR 10.5) than those without delirium (3.9 IQR 7.9). More than three quarter of patients with delirium were in the high or intermediate HFRS group. Patients with delirium had significantly higher in‐hospital mortality (7.6% vs. 6.6%), 30‐day mortality (13.3% vs. 4.4%), 30‐day readmission (15.3% vs. 8.0%), median LOS (7.0 IQR 7.0 vs. 4.0 IQR 5.0) and median total cost (SGD$5289 IQR SGD$4921 vs. SGD$3816 IQR SGD$3855). All the allied health cost for physiotherapy, occupational therapy, and dietitian was significantly higher in patients with delirium (*p*=<0.001). Similarly, the breakdown cost for laboratory, radiology and medications costs were also significantly higher in persons with delirium.

**FIGURE 1:**
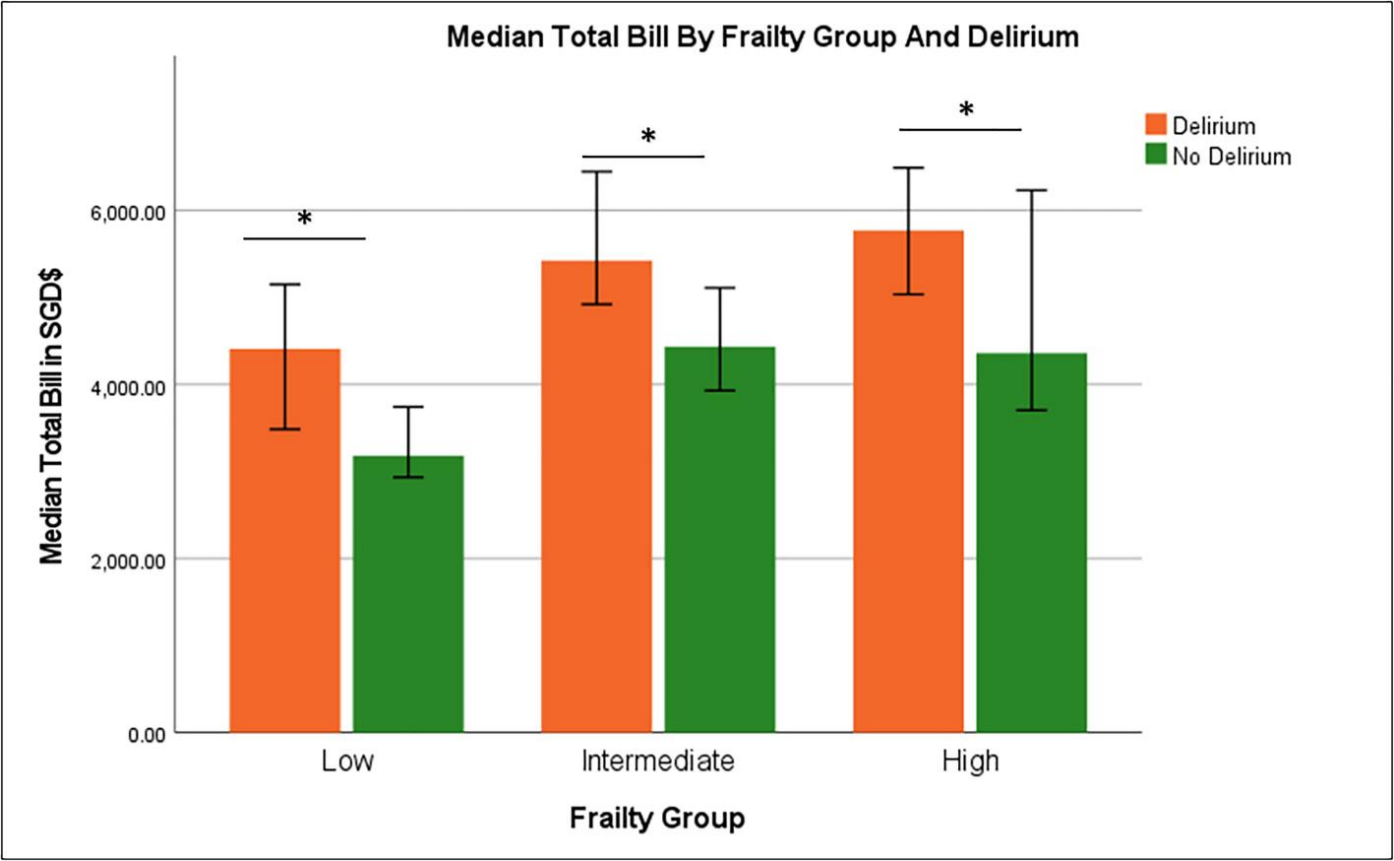
Median total bill by frailty group and delirium.

**FIGURE 2:**
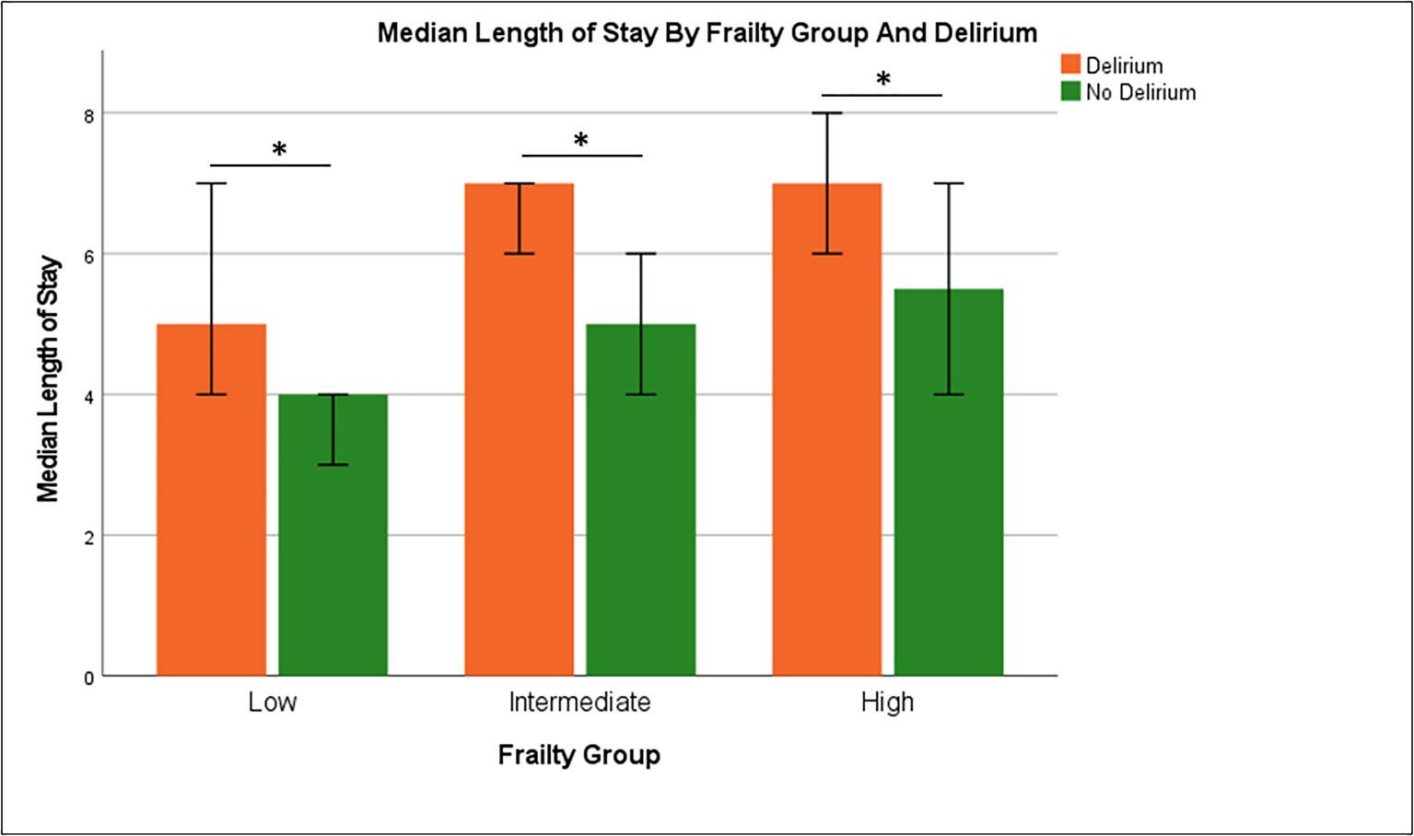
Median length of stay (LOS) by frailty group and delirium.

In the adjusted multivariate analysis for association of delirium with clinical diagnosis and comorbidities (Table 2), delirium was significantly associated with at least intermediate frailty (OR 3.51, 95% CI 2.48–4.98), dementia (OR 2.39, 95% CI 1.61–3.54), UTI (OR 1.95, 95% CI 1.29–2.95), constipation (OR 2.49, 95% CI 1.43–4.33), *Klebsiella* infection (OR 3.06, 95% CI 1.28–7.30) and dehydration (OR 2.01, 95% CI 1.40–2.88). In the fully adjusted model for healthcare utilisation indices and mortality (Table 3), delirium was significantly associated with higher 30‐days mortality (OR 2.52; CI 1.42–4.47), 30‐day readmission (OR 2.18; CI 1.36–3.48), eLOS (OR 1.80; CI 1.30–2.49) and eCOST (OR 1.67; CI 1.20–2.35) independent of dementia. Delirium was significantly associated with increased total cost (Figure 1) and increased LOS across the frailty groups (Figure 2).

**TABLE 2:** Association of delirium with discharge diagnosis and comorbidities

Predictors	Unadjusted OR (95% CI) *p* value	Adjusted OR (95% CI) *p* value
Age ≥80 years	**1.582 (1.112–2.251) 0.011**	1.252 (0.823–1.903) 0.294
At least intermediate frailty	**5.241 (3.851–7.133) <0.001**	**3.513 (2.476–4.984) <0.001**
Age adjusted Charlson comorbidity index >6	**2.058 (1.553–2.729) <0.001**	1.422 (0.894–2.262) 0.137
Dementia	**3.493 (2.497–4.886) <0.001**	**2.386 (1.607–3.544) <0.001**
Diabetes mellitus	**1.349 (1.028–1.770)** **0.031**	0.772 (0.451–1.320) 0.344
Hypertension	**1.333 (1.017–1.746) 0.037**	1.084 (0.725–1.622) 0.694
Hyperlipidaemia	**1.407 (1.064–1.861) 0.017**	1.437 (0.863–2.393) 0.163
Chronic kidney disease	**1.656 (1.257–2.181) <0.001**	0.955 (0.648–1.409) 0.818
Intracranial haemorrhage	2.111 (0.880–5.064) 0.094	1.699 (0.648–4.455) 0.281
Stroke	**6.959 (1.969–24.598) 0.003**	3.208 (0.815–12.633) 0.096
Acute myocardial infarction	**1.818 (1.141–2.898) 0.012**	1.407 (0.798–2.480) 0.238
Pneumonia	**1.783 (1.336–2.380) <0.001**	1.316 (0.919–1.884) 0.134
Urinary tract infection	**2.962 (2.134–4.112) <0.001**	**1.949 (1.288–2.949) 0.002**
Acute retention of urine	1.500 (0.984–2.285) 0.059	0.927 (0.559–1.538) 0.769
Constipation	**2.482 (1.550–3.974) <0.001**	**2.489 (1.430–4.334) 0.001**
*Klebsiella* infection	**3.582 (1.676–7.660)** **<0.001**	**3.060 (1.282–7.302) 0.012**
*E Coli* infection	**1.914 (1.295–2.829) 0.001**	1.093 (0.666–1.794) 0.725
Dehydration	**2.813 (2.077–3.809) <0.001**	**2.011 (1.403–2.883) <0.001**
Hyponatremia	1.288 (0.888–1.868) 0.182	0.961 (0.617–1.499) 0.862
Fragility fracture	0.796 (0.420–1.506) 0.483	1.422 (0.894–2.262) 0.137

*Note*: Bold values are statistically significant.

**TABLE 3:** Association of delirium with readmission within 30 days, in‐hospital mortality, cost and length of stay (LOS) (Non‐delirious as reference group)

Predictors	Unadjusted OR (95% CI) *p* value	Adjusted OR (95% CI) *p* value
In‐hospital mortality	1.180 (0.703–1.981) 0.531	0.956 (0.532–1.718) 0.882
30‐day mortality	**3.432 (2.054–5.734) <0.001**	**2.524 (1.424–4.473) 0.002**
Readmission within 30 days	**2.073 (1.358–3.164) <0.001**	**2.175 (1.360–3.479) 0.001**
eLOS	**2.398 (1.785–3.223) <0.001**	**1.795 (1.296–2.487) <0.001**
eCOST	**2.178 (1.603–2.958) <0.001**	**1.674 (1.193–2.349) 0.003**

*Note*: Adjusted for age, frailty status, dementia and age associated Charlson Comorbidity Index. Bold values are statistically significant.

## 4 DISCUSSION

This study systematically assessed the association between delirium, frailty defined by HFRS, dementia, comorbidities and common admission diagnoses such as pneumonia, fragility fractures, acute retention of urine, UTI, constipation, hyponatremia, dehydration, stroke, intracranial bleed, acute myocardial infarction, *E. coli* infection and *Klebsiella* infection in older inpatients. The prevalence of patients with stroke, intracranial bleed and acute myocardial infarction in our study population was relatively low as most of the patients would have been admitted to the relevant specialities if diagnosis was made at the emergency department. The admission criteria to the Division of Geriatric Medicine is based on age ≥75 years old with six attending geriatricians all trained within the same tertiary centre. The diagnosis of delirium was made by the attending geriatrician based on the Confusion Assessment Method of acute onset and fluctuating mental status, inattention and either altered level of consciousness or disorganized thought.^19^ The prevalence of delirium in our study was 39.1%, which was similar to other studies where it is reported be between 13% and 42% at admission, 26%–62% during hospitalisation, and increasing to 90% in the weeks leading to death.^4^, ^20^, ^21^ Delirium was significantly associated with higher 30‐days mortality, 30‐days readmission, eLOS and eCOST.

In older patients, multiple pathologies are common, and delirium is caused by multiple interacting factors which require simultaneous multicomponent management. There was significantly higher prevalence of chronic diseases such as diabetes, hypertension, hyperlipidaemia, chronic kidney disease and dementia in older patients with delirium. Similarly, the diagnosis of infections such as pneumonia, UTI, *E. coli* and *Klebsiella* infections and other diagnoses such as constipation, dehydration, stroke, intracranial bleed and acute myocardial infarction were also significantly higher in patients with delirium. In the fully adjusted model, at least intermediate frailty, pneumonia, UTI, constipation, *Klebsiella* infection and dehydration remained significant. While Monacelli et al only found neurosensorium multimorbidity such as dementia, cerebrovascular diseases and sensory impairment to be significantly more prevalent in patients with delirium,^22^ hyperlipidaemia, hypertension and diabetes are known risk factors for cardiovascular disease, stroke disease and dementia. Dementia and stroke are in itself are risk factors for delirium.

Frailty and delirium are intrinsically connected where the inflammatory cytokine cascade is hypothesised to trigger neuroinflammation and disruption of large‐scale neuronal networks in the brain, causing acute functional and cognitive decline.^23^, ^24^ Vulnerability in frailty is thought to be due to lifelong accumulation of physical and cognitive deficits which predisposes frail older patients to delirium.^25^ In the same vein, delirium is a reflection of acute decompensation of underlying frailty in an older adult.^26^ Both frailty and delirium are independently associated with increased mortality in hospitalised older adults.^27^

Delirium, but not age or Charlson Comorbidity Index, was significantly associated with at least intermediate frailty in our study population. While previous studies used traditional measures of frailty which can be resource intensive and subject to interrater error, HFRS can be auto‐populated from routine electronic medical records.^10^, ^28^ The diagnosis of delirium is often missed by healthcare professionals. Multicomponent intervention has been shown to prevent delirium in 40% of older inpatients, although it may not necessarily change the overall trajectory of delirium in patients with existing delirium.^7^ There is potential that electronic medical record alerts raised through HFRS scores could mitigate the underdiagnosis of delirium, and facilitate early preventive interventions when paired with recommended multicomponent checklists and pathways.

While delirium in known to be associated with severity of illness, the significant association of *Klebsiella* infection with delirium has not been reported before. *Klebsiella* is one of the major pathogens responsible for localised infection such as pneumonia and UTI, abscesses and bacteraemia. Reported mortality for *Klebsiella pneumoniae* bacteraemia in Singapore is between 20% and 26%.^29^ Those associated with disseminated infections and liver abscesses are often referred to as hypervirulent *Klebsiella pneumoniae* which is found in countries like Singapore, Taiwan and South Korea.^30^ Hypervirulent *Klebsiella pneumoniae* can also cause meningitis and endogenous endophthalmitis.^29^ It is not known if the significant association of *Klebsiella* infection with delirium is unique to Asia Pacific countries due to higher prevalence of the hypervirulent *Klebsiella pneumoniae* strain.

Urinary tract infection, retention of urine, dehydration and constipation are well known precipitating factors for delirium in published literature. Constipation was present in 13.3% of our older inpatients while prevalence in the published literature ranges between 14% and 40% depending on the population studied.^31^ While constipation in itself can be a cause for delirium, factors associated with constipation such as dehydration, immobility, anticholinergic medications load or poorly controlled diabetes mellitus can also independently precipitate delirium. Dehydration can cause postural hypotension with decrease in cerebral perfusion. In addition, our brain consists of 80% of water with majority of the central nervous system intracellular water being stored in astrocytes. The effect of dehydration results in upregulation and overexpression of aquaporins in these astrocytes, resulting in extracellular dehydration and hence delirium.^32^ Studies have shown that loss of 1%–2% of total body water can lead to cognitive impairment.^32^ Factors causing dehydration such as inability to access water due to various reasons, cognitive impairment, excess diuretics, or swallowing impairment need to be addressed in a person with delirium due dehydration. While urinary retention was not significantly more prevalent in our study group with delirium, it is known to precipitate delirium possibly through increased sympathetic stimulation and catecholamine surge from bladder distension and known as cystocerebral syndrome.^33^

Dementia was significantly associated with delirium, which is a well‐known phenomenon.^9^ The prevalence of dementia in our study population may be higher as delirium and dementia often co‐exist, however, the DSM‐5 criteria explicitly states that dementia cannot be diagnosed in the presence of delirium.^34^ Dementia and delirium share a bidirectional relationship with similar pathophysiology including inflammation, cholinergic deficit, mitochondrial dysfunction, impaired cerebral blood flow and metabolism with neuronal death.^35^ Recurrent delirium can accelerate a decline in dementia especially in the frail older patient, which makes delirium prevention a top priority one in older hospitalised patients with dementia. The association of delirium with 30‐day mortality, readmission, eLOS and eCOST was independent of underlying dementia.

The total hospitalization cost of patients with delirium was 1.4x higher compared to those without delirium in our study. These patients were managed by geriatricians who routinely provide comprehensive geriatric assessment and multidisciplinary care. Similarly, LOS for patients with delirium was 1.8x longer. We have no data for comparison locally as delirium is known to be under‐diagnosed by healthcare professionals and the above data is dependent on accuracy of coding. It is well known that delay in the diagnosis of delirium is associated with adverse outcomes and mortality although there is limited data available in the acute inpatient setting.^36^ In a recent scoping review, there was a wide variation in increased cost associated with delirium ranging from $1532 to $22,269 depending on the country, patient population and healthcare systems. Similarly, increased LOS varied between 2.5 and 10.4 additional days.^37^

Significantly worse outcomes were observed for patients with delirium in our study, with nearly twice the 30‐days mortality rate and a 1.2‐fold higher 30‐days readmission rate as compared to those without delirium. Delirium has been described as an independent marker for increased mortality among older inpatients up to 12 months post‐discharge and shown to be a significant predictor of 30‐days hospital readmission.^23^ Strategies to reduce readmissions associated with delirium will have to be targeted at reducing the incidence of hospital‐acquired delirium and providing adequate caregiver support during the transitional period post‐discharge.

Our study has multiple strengths including large sample size and robust database. A limitation of this study was that the diagnosis of delirium, although based on confusion assessment method, was dependent on clinicians judgement and they did not undergo a standardised training with a checklist. We did not have information on the duration of delirium, and if it was present on admission or developed during inpatient stay. Due to the retrospective nature of the study, information on pain, vision impairment, hearing impairment, burden of anticholinergic medications, baseline cognition, function and psychosocial situation, which are also predictive of delirium^38^ was not available. Data on mortality and readmissions were only available within the single institution. Hospital Frailty Risk Score does not capture the fluctuations caused by acute illness and hence is not an accurate measure of function. Lastly, accuracy of the data obtained is dependent on the accuracy of the coding, and no causal inferences can be made from the association.

Our study highlighted a few significant findings and is one of the first few to document cost of delirium. Firstly, there was significant association between HFRS and delirium where more than three quarters of patients with delirium were classified under the intermediate and high frailty group. Hospital Frailty Risk Score can be auto‐populated from electronic medical records and is a feasible tool for risk stratification in the acute care setting. However, one in five delirious patients may be identified as low frailty and hence may be missed. Future studies will need to evaluate the association of delirium with composite scores of HFRS and Modified Early Warning Scores as a measure of illness severity which is available in electronic health records.^39^ Frailty and dementia have been well‐known to be significant risk factors for disability, for which both were significantly associated with delirium in our study. Frailty, dementia and delirium share a triangular relationship where frailty contributes to the risk for dementia and delirium, while recurrent delirium accelerates dementia and/or frailty onset and decline. Of note, frailty may be reversible through interventions at population level.^40^ It should be every hospital leader's priority to implement measures to prevent delirium in high‐risk groups and manage frailty and delirium in tandem to reduce overall cost and LOS.

## 5 CONCLUSION

Delirium is highly prevalent in older inpatients, and associated with increased cost, LOS, 30‐days readmissions and mortality. Significantly, frailty and dementia were strongly associated with delirium as compared to chronological age or multimorbidity. The HFRS which can be auto‐populated was significantly associated with delirium and could serve as a practical tool for widespread implementation in healthcare systems to identify vulnerable older adults at risk of delirium. Prospective studies are needed to analyse the benefits of multicomponent delirium preventive measures in the high‐risk groups identified by HFRS scores within the acute care setting.

## CONFLICT OF INTEREST

The authors have no conflicts of interest to declare.

## Data Availability

The data that support the findings of this study are available on request from the corresponding author. The data are not publicly available due to privacy or ethical restrictions.
